# Drug utilization of paliperidone in adolescent schizophrenia patients: A retrospective cohort study in China

**DOI:** 10.1192/j.eurpsy.2021.1368

**Published:** 2021-08-13

**Authors:** S. Dong, T. Zhang, T. Wu, L. Zhang, H. Sun, W. Dong, H. Wang

**Affiliations:** 1 Epidemiology, Janssen Research and Development, China, Shanghai, China; 2 Epidemiology, Janssen Research and Development, Beijing, China; 3 Medical Affairs, Janssen Research and Development, China, Shanghai, China; 4 Peking University The Sixth Hospital, Institute Of Mental Health; National Clinical Research Center For Mental Health Disorders & Key Laboratory Of Mental Health, Ministry Of Health, Peking University, Peking University the Sixth Hospital, Beijing, China; 5 Xijing Hospital, the Fourth Military Medical University, Xi’an, China, xian, China

**Keywords:** Drug Utilization, paliperidone, schizophrénia, adolescent

## Abstract

**Introduction:**

In China, the indications of paliperidone extended in schizophrenia adolescents (12-17 years) was approved by National Medical Products Administration (NMPA) in 2017. But, the utilization of paliperidone in this group needs to be further investigated.

**Objectives:**

To assess paliperidone utilization in schizophrenia adolescents.

**Methods:**

The study employed the electronic medical records (EMRs) database from a psychiatry specialized hospital (PH) and a general hospital (GH), respectively. General information, including birth date, gender, visit date, diagnosis (inpatient and outpatient) with ICD-10 coding, drug characterize, prescription date and dosage, was de-identified and standardized for analysis. Schizophrenia adolescents (ICD-10: F20.x) received at least one prescription of paliperidone between 2018 and 2019 were included in this study. Index date was defined as the date of first identified paliperidone prescription. The patients were followed up until the end of 2019 with the last record, or upon reaching 18 years. The database was analyzed based on days of supply, administration frequency, and daily dose.

**Results:**

Overall, 112 and 117 eligible patients were included in the present study from PH and GH, respectively. The median drug supply was 179.0 days and 44.0 days, respectively, during which median number of prescriptions patients received was 6.0 and 3.0. Paliperidone was mostly initiated alone (57.1% and 88.9%) with frequency of once daily (97.3% and 88.9%), and the median of average daily dose during follow-up was 5.7 mg/day and 6.0 mg/day, respectively.

**Conclusions:**

The duration of paliperidone usage was very different in two hospitals, but the dosages in both hospitals were generally agreed with prescribing information.

